# Nomogram based on radiomics analysis of ultrasound images can improve preoperative *BRAF* mutation diagnosis for papillary thyroid microcarcinoma

**DOI:** 10.3389/fendo.2022.915135

**Published:** 2022-08-19

**Authors:** Jiajia Tang, Shitao Jiang, Jiaojiao Ma, Xuehua Xi, Huilin Li, Liangkai Wang, Bo Zhang

**Affiliations:** ^1^ Chinese Academy of Medical Sciences and Peking Union Medical College, Beijing, China; ^2^ Department of Ultrasound, China-Japan Friendship Hospital, Beijing, China; ^3^ Department of Liver Surgery, Peking Union Medical College Hospital, Chinese Academy of Medical Sciences and Peking Union Medical College, Beijing, China; ^4^ Institute of Clinical Medicine, China-Japan Friendship Hospital, Beijing, China; ^5^ Department of Ultrasound, China-Japan Friendship Hospital, National Center for Respiratory Medicine, National Clinical Research Centerfor Respiratory Diseases, Institute of Respiratory Medicine of Chinese Academy of Medical Sciences, Beijing, China

**Keywords:** Nomogram, ultrasound (US), radiomics, *BRAF* mutation, papillary thyroid microcarcinoma (PTMC)

## Abstract

**Background:**

The preoperative identification of *BRAF* mutation could assist to make appropriate treatment strategies for patients with papillary thyroid microcarcinoma (PTMC). This study aimed to establish an ultrasound (US) radiomics nomogram for the assessment of *BRAF* status.

**Methods:**

A total of 328 PTMC patients at the China-Japan Friendship Hospital between February 2019 and November 2021 were enrolled in this study. They were randomly divided into training (*n* = 232) and validation (*n* = 96) cohorts. Radiomics features were extracted from the US images. The least absolute shrinkage and selection operator (LASSO) regression was applied to select the *BRAF* status-related features and calculate the radiomics score (Rad-score). Univariate and multivariate logistic regression analyses were subsequently performed to identify the independent factors among Rad-score and conventional US features. The US radiomics nomogram was established and its predictive performance was evaluated *via* discrimination, calibration, and clinical usefulness in the training and validation sets.

**Results:**

Multivariate analysis indicated that the Rad-score, composition, and aspect ratio were independent predictive factors of *BRAF* status. The US radiomics nomogram which incorporated the three variables showed good calibration. The discrimination of the US radiomics nomogram showed better discriminative ability than the conventional US model both in the training set (AUC 0.685 vs. 0.592) and validation set (AUC 0.651 vs. 0.622). Decision curve analysis indicated the superior clinical applicability of the nomogram compared to the conventional US model.

**Conclusions:**

The US radiomics nomogram displayed better performance than the conventional US model in predicting *BRAF* mutation in patients with PTMC.

## Introduction

Papillary thyroid cancer (PTC) is the main contributor to the rapidly rising incidence of thyroid malignancies worldwide (1). Approximately 40%–50% of PTC is composed of papillary thyroid microcarcinomas (PTMCs), which are defined as the maximum diameter of tumors less than or equal to 10 mm (2). Although the majority of PTMCs are known to have indolent disease progression and excellent outcomes (3), some patients with high-risk features do have aggressive tumor behavior, including local recurrence and metastasis (4). For clinically low-risk PTMC patients, defined as the absence of extrathyroidal extension (ETE), lymph node metastasis (LNM), and distant metastasis, the standard management varies differently in clinical practice, and active surveillance has emerged as an alternative to surgical resection (3, 5). Nevertheless, some patients with clinically low-risk PTMC still show recurrence in the later course, resulting in a poor prognosis.

*BRAF* mutation is the most common genetic alteration in PTC (6), which has been proven to drive aggressive tumor behavior. Abundant studies revealed that *BRAF* mutation was independently correlated with recurrence and metastasis of PTMC, which can be introduced as a genetic prognostic indicator for PTMC patients (5, 7–9). In that case, the preoperative diagnosis of the *BRAF* mutation can help identify high-risk PTMC patients and plan more aggressive treatment strategies.

Fine-needle aspiration (FNA) is the most commonly used technique to diagnose *BRAF* mutation in preoperative evaluation (10). However, current guidelines do not recommend FNA for nodules with a diameter less than 10 mm (11–13). Although some patients presented with high-risk thyroid nodules on ultrasound, they would not be suggested to do the FNA due to the small diameter of the nodules. Some patients might choose conservative surveillance and still have unclear *BRAF* mutation status. Therefore, if *BRAF* mutation status can be determined in the preoperative examination, further FNA and gene sequencing could provide more critical information for risk stratification and surgical planning.

Ultrasound (US) is the primary imaging technique for the evaluation of thyroid nodules (14). Several studies have investigated whether conventional US features could predict the presence of the *BRAF* mutation in PTMC and have reported controversial results, which might be due to limitations of the conventional US image, such as high dependency on the radiologist’s experience and interobserver variation (15). Radiomics, which automatically extracts innumerable high-dimensional features from images, has recently emerged as a novel method to acquire more information from image material. Radiomics analyses based on US images have been utilized to predict molecular properties in various cancers, including PTC (16, 17). To our knowledge, there are no published studies aimed at identifying the presence of *BRAF* mutation in PTMC using US radiomics features. Therefore, this study was conducted in order to explore the association between *BRAF* mutation status and radiomics features and develop a US radiomics nomogram for the prediction of preoperative *BRAF* mutation for PTMC patients.

## Materials and methods

### Patient selection

Between February 2019 and November 2021, consecutive patients from the China-Japan Friendship Hospital were included. This retrospective study was approved by the Institutional Review Board of China-Japan Friendship Hospital. The inclusion criteria of this study were detailed as follows: 1) the target nodule was pathologically confirmed as PTC; 2) the largest size of the target nodule was less or equal to 10 mm; 3) the US image of the targeted tumor in the longest axis was available; and 4) the *BRAF* mutation test was performed and acquired a specific diagnostic result. The exclusion criteria were as follows: 1) the pathological and *BRAF* mutation status of the target tumor was uncertain; 2) the patient previously underwent thyroidectomy, radiofrequency ablation, or radiotherapy; and 3) concomitant with other types of thyroid malignancies or other variants of gene mutation. The final analyzed cohort consisted of 328 patients with PTMC. According to the gene mutation status, the patients were divided into two groups: BRAF (+) and BRAF (−). By using the “createDataPartiton” function in R (version 3.6.3), the discovery cohort was divided into training and validation sets with a ratio of 7:3. All the included patients were finally classified into training (*n* = 232) and validation (*n* = 96) cohorts. Patients’ information about age, gender, and conventional US features was collected from medical records.

### *BRAF* mutation analysis

US-guided FNAs were performed by an experienced radiologist with more than 20 years of experience in FNA for thyroid nodules. At least three repeated aspirations were performed in different directions. Samples obtained with 23-gauge needles were sent for cytological examination. The remaining tissue was flushed into normal saline and used for *BRAF* ^V600E^ mutation analysis. Genomic DNA was extracted from FNA specimens using AmoyDx^®^ *BRAF* Mutation Detection Kit (V2) (ADx-BR02) provided by Amoy Diagnostics Co., Ltd. (Xiamen, China). Mutations of *BRAF* ^V600E^ were analyzed by a next-generation sequencing (NGS) method. This was followed by a real-time fluorescence polymerase chain reaction-amplification refractory mutation system (PCR-ARMS).

### US image acquisition and feature evaluation

US examinations were performed by radiologists with 5–20 years of experience in thyroid US evaluation. These examinations were performed with a 5–14-MHz transducer (Siemens, ACUSON Sequoia, Siemens Medical Solutions USA, Inc. Malvern, USA). US images were stored digitally as Digital Imaging and Communications in Medicine (DICOM) in axial and/or sagittal planes of the thyroid nodules for subsequent evaluation.

All the US images were independently reviewed by two experienced radiologists to assess the US features in a single-blind manner. They did not know whether there was *BRAF* mutation of the target nodule when evaluating the US features. The two radiologists made final decisions by consensus when discordant cases occurred. The conventional US features include shape (regular, irregular), margin (smooth, rough), aspect ratio (≥1 or<1), calcification (absent or present), halo (absent or present), location (right lobe, left lobe, isthmus), maximum diameter of the tumor, association with the capsule (subcapsule thyroidal or intrathyroidal), and close to the tracheal cartilage (absent or present). The vascular characteristics of the target tumor were classified into five types by Frates et al. (18): 0 for no visible flow, 1 for minimal internal flow without a peripheral ring, 2 for a peripheral ring of flow (defined as >25% of the nodule’s circumference) with minimal or no internal flow, 3 for a peripheral ring of flow with a small to moderate amount of internal flow, and 4 for extensive internal flow with or without a peripheral ring.

### Feature selection and radiomics score establishment

The MaZda software (version 4.6, available at http://www.eletel.p.lodz.pl/mazda/) was used for texture analysis (TA) (19). The region of interest (ROI) was manually delineated on the largest diameter image using the MaZda software. All the manual segmentations were conducted by two radiologists and one radiologist (twice) with more than 4 years of experience in thyroid imaging who were blinded from the *BRAF* mutation status (for interobserver and intraobserver reproducibility evaluation). The interclass correlation coefficient (ICC) was used to evaluate the interobserver and intraobserver agreement of the feature extraction. An ICC larger than 0.80 was considered excellent. The MaZda software allows the computation of almost 298 radiomics features, which were classified into six categories: (1) HISTOGRAM (2) GRADIENT (3) RUN LENGTH MATRIX (4) CO-OCCURRENCE MATRIX (5) AUTOREGRESSIVE MODEL (6) HAAR WAVELET. The list of radiomics features is summarized in **Supplementary Table 1**.

Feature selection and building radiomics score (Rad-score) were based on the training cohort. The least absolute shrinkage and selection operator (LASSO) logistic regression method using 10-fold cross-validation was applied to select the most useful predictive *BRAF*-related features from the training cohort (20). The Rad-score was generated using a linear combination of the selected features weighted by the LASSO algorithm.

### Development of the US radiomics nomogram

**Figure 1:**
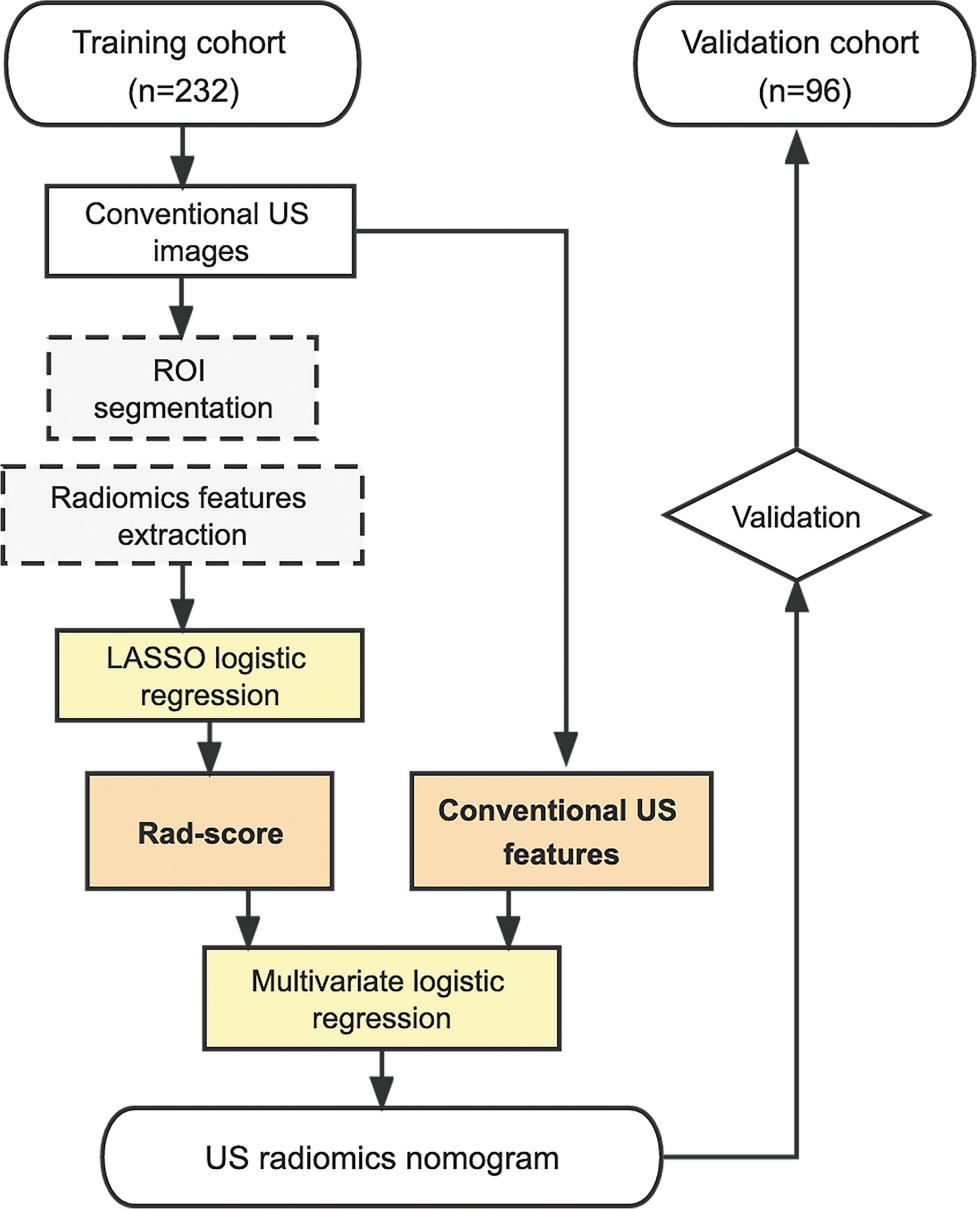
Procedure of patient selection and radiomics nomogram development. US, ultrasound; ROI, region of interest; LASSO, least absolute shrinkage and selection operator; Rad-score, radiomics score.

Univariate analyses, consisting of Student’s independent *t*-test (continuous variables) and *χ* ^2^ test (categorical variables), were used to select the risk indicators associated with *BRAF* mutation. A multivariate logistic regression analysis incorporating Rad-score and conventional US variables was performed, using the backward step-down selection procedure with a liberal *P <*0.05 as the retention criteria to select the final indicators for *BRAF* mutation. Then, a US radiomics nomogram was developed based on the multivariate analysis in the training cohort. For comparison, a conventional US model was developed using the independent conventional US features alone without Rad-score. The whole procedure of the patient enrollment and model development is illustrated in **Figure 1**.

### Predictive performance of the US radiomics nomogram

Calibration of the US radiomics nomogram was evaluated using the calibration curve. The discrimination performance of the nomogram was evaluated using the area under the receiver operating characteristic (ROC) curve (AUC). Decision curve analysis (DCA) was conducted to determine the clinical utility of the US radiomics nomogram by quantifying the net benefits at different threshold probabilities. The calibration, discrimination, and clinical utility of the nomogram were verified in the validation cohort. For clinical application, the predicted probability (defined as Nomo-score) of each patient was calculated according to the nomogram algorithm. Then, the optimal cutoff value was determined by maximizing the Youden index. The performance of the optimal cutoff value of the Nomo-score was assessed by the AUC, as well as sensitivity, specificity, positive predictive value (PPV), and negative predictive value (NPV).

### Statistical analysis

Descriptive statistics are described as median (interquartile range, IQR) for categorical variables and frequency (%) for categorical variables. Pearson’s chi-square or Fisher’s exact test was used to compare differences for categorical characteristics. The independent sample *t*-test was performed for continuous factors with normal distribution, whereas the Mann–Whitney *U* test was used for continuous factors without normal distribution. Bonferroni-adjusted significance tests were applied for pairwise comparisons. All statistical analyses were performed with R 3.6.3 software (http://www.r-project.org). The statistical tests were two-sided, with statistical significance indicated by *P*-values lower than 0.05.

## Results

### Baseline characteristics

A total of 328 patients with PTMC were enrolled with an average age of 39 (IQR: 33 to 47) years and a male-to-female ratio of 87:241. Patients’ clinical and conventional US characteristics in the training and validation cohorts are summarized in **Table 1**. There were no differences in clinical and US characteristics between the two cohorts (*P* > 0.05). *BRAF*-positive [*BRAF* (+)] accounted for 72.4% and 70.8% of the training and validation cohorts, respectively. *BRAF*-negative [*BRAF* (−)] accounted for 27.6% and 29.2% of the training and validation cohorts, respectively. There were no differences in *BRAF* mutation status between the two cohorts (*P* = 0.877).

**Table 1:** Clinical and US characteristics of PTMC patients in the training and validation cohorts.

Variables	Training cohort (*n* = 232)	Test cohort (*n* = 96)	*P-*value
*BRAF* mutation			0.877
Negative	64 (27.6%)	28 (29.2%)	
Positive	168 (72.4%)	68 (70.8%)	
Age, median (IQR), years	39 (33, 47)	39.5 (33, 48)	0.887
Gender			0.267
Female	175 (75.4%)	66 (68.8%)	
Male	57 (24.6%)	30 (31.2%)	
Multifocality			0.518
Negative	187 (80.6%)	81 (84.4%)	
Positive	45 (19.4%)	15 (15.6%)	
Primary site			0.776
Right lobe	119 (51.3%)	46 (47.9%)	
Left lobe	99 (42.7%)	45 (46.9%)	
Isthmus	14 (6%)	5 (5.2%)	
Tumor location			0.887
Intrathyroidal	139 (59.9%)	56 (58.3%)	
Subcapsular thyroid	93 (40.1%)	40 (41.7%)	
Tumor size (mm)			0.889
≤5	59 (25.4%)	23 (24%)	
5–10	173 (74.6%)	73 (76%)	
Composition			0.749
Mixed cystic and solid	16 (6.9%)	5 (5.2%)	
Solid	216 (93.1%)	91 (94.8%)	
Homogeneity			0.875
Homogeneous	86 (37.1%)	34 (35.4%)	
Heterogeneous	146 (62.9%)	62 (64.6%)	
Aspect ratio ≥1			0.139
Absent	66 (28.4%)	36 (37.5%)	
Present	166 (71.6%)	60 (62.5%)	
Calcification			0.356
Absent	104 (44.8%)	37 (38.5%)	
Present	128 (55.2%)	59 (61.5%)	
Close to the trachea cartilage			0.720
Absent	194 (83.6%)	78 (81.2%)	
Present	38 (16.4%)	18 (18.8%)	
Vascular type			0.440
Type 0	34 (14.7%)	7 (7.3%)	
Type 1	54 (23.3%)	22 (22.9%)	
Type 2	58 (25%)	29 (30.2%)	
Type 3	32 (13.8%)	14 (14.6%)	
Type 4	54 (23.3%)	24 (25%)	
Rad-score, median (IQR)	0.97 (0.6, 1.32)	0.88 (0.61, 1.24)	0.519

US, ultrasound; PTMC, papillary thyroid microcarcinoma; Rad-score, radiomics score; IQR, interquartile range.

### Establishment of the US radiomics score

**Figure 2:**
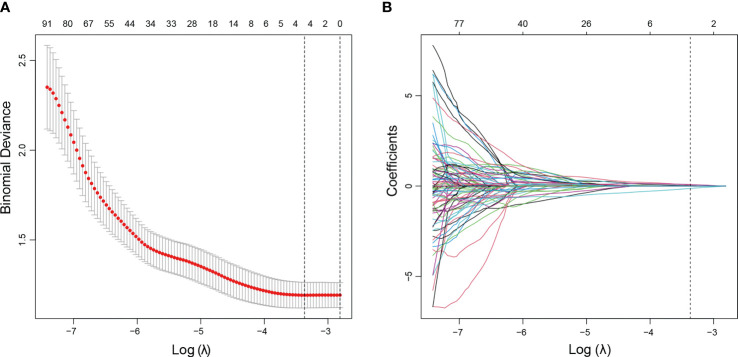
US radiomics feature selection using LASSO logistic regression model in the training cohort. **(A)** The tuning parameter (*λ*) was selected using 10-fold cross-validation *via* minimum criteria. Dotted vertical lines were drawn at the optimal values using the minimum criteria and 1 standard error of the minimum criteria (1−SE criteria). A *λ* value of 0.0273, with a log (*λ*) value of −3.601, was obtained. **(B)** The vertical line was drawn at the value selected using 10-fold cross-validation, where optimal *λ* resulted in four non-zero coefficients. LASSO, least absolute shrinkage and selection operator.

The nodule under FNA and *BRAF* mutation was selected for analysis. For patients with more than one nodule, only the one under FNA and *BRAF* mutation was applied. The ROI was manually drawn using the MaZda software for all the target tumors. Favorable interobserver and intraobserver reproducibility of feature extraction was achieved, with intraobserver ICCs ranging from 0.783 to 0.997 and the interobserver ICCs ranging from 0.792 to 0.984. A total of 298 radiomics features were extracted from each US image. The LASSO logistic regression method using 10-fold cross-validation was applied to select the most useful predictive *BRAF*-related features from the training cohort. A total of 298 radiomics features were reduced to four potential predictors by the LASSO regression model (**Figure 2**). The Rad-score calculation formula was presented as follows. The detailed description of parameters in the formula is shown in **Supplementary Table 1**. The Rad-scores were much higher in the *BRAF* (+) group in both the training and validation sets than those in the *BRAF* (−) group (**Table 2**).

**Table 2:** Demographic and sonographic characteristics of PTMC patients by *BRAF* mutation status in the training cohort (*n* = 232).

Variables	*BRAF* (−) (*n* = 64)	*BRAF* (+) (*n* = 168)	*P*-value
Gender			0.149
Female	53 (82.8%)	122 (72.6%)	
Male	11 (17.2%)	46 (27.4%)	
Multifocality			0.477
Negative	54 (84.4%)	133 (79.2%)	
Positive	10 (15.6%)	35 (20.8%)	
Primary site			0.864
Right lobe	32 (50%)	87 (51.8%)	
Left lobe	29 (45.3%)	70 (41.7%)	
Isthmus	3 (4.7%)	11 (6.5%)	
Tumor location			0.729
Intrathyroidal	40 (62.5%)	99 (58.9%)	
Subcapsular thyroid	24 (37.5%)	69 (41.1%)	
Tumor size (mm)			0.549
≤5	14 (21.9%)	45 (26.8%)	
5–10	50 (78.1%)	123 (73.2%)	
Composition			0.046*
Mixed cystic and solid	8 (12.5%)	8 (4.8%)	
Solid	56 (87.5%)	160 (95.2%)	
Homogeneity			0.499
Homogeneous	21 (32.8%)	65 (38.7%)	
Heterogeneous	43 (67.2%)	103 (61.3%)	
Aspect ratio ≥1			0.018*
Absent	26 (40.6%)	40 (23.8%)	
Present	38 (59.4%)	128 (76.2%)	
Calcification			0.955
Absent	28 (43.8%)	76 (45.2%)	
Present	36 (56.2%)	92 (54.8%)	
Close to the trachea cartilage			0.696
Absent	55 (85.9%)	139 (82.7%)	
Present	9 (14.1%)	29 (17.3%)	
Vascular type			0.087
Type 0	12 (18.8%)	22 (13.1%)	
Type 1	9 (14.1%)	45 (26.8%)	
Type 2	14 (21.9%)	44 (26.2%)	
Type 3	8 (12.5%)	24 (14.3%)	
Type 4	21 (32.8%)	33 (19.6%)	
Age, median (IQR)	39 (32.75, 46.25)	39 (34.75, 47)	0.585
Rad-score, median (IQR)	0.76 (0.51, 1.01)	1.04 (0.67, 1.39)	<0.001

PTMC, papillary thyroid microcarcinoma; Rad-score, radiomics score; IQR, interquartile range. *p < 0.05.

Radiomics score (Rad-score) = 0.49815794 * Horzl_LngREmph + 0.32953014 * GrSkewness − 0.40707617 * Teta1 + 0.00094667 * Teta2 + 1.05750093.

### Development and validation of the US radiomics nomogram

**Figure 3:**
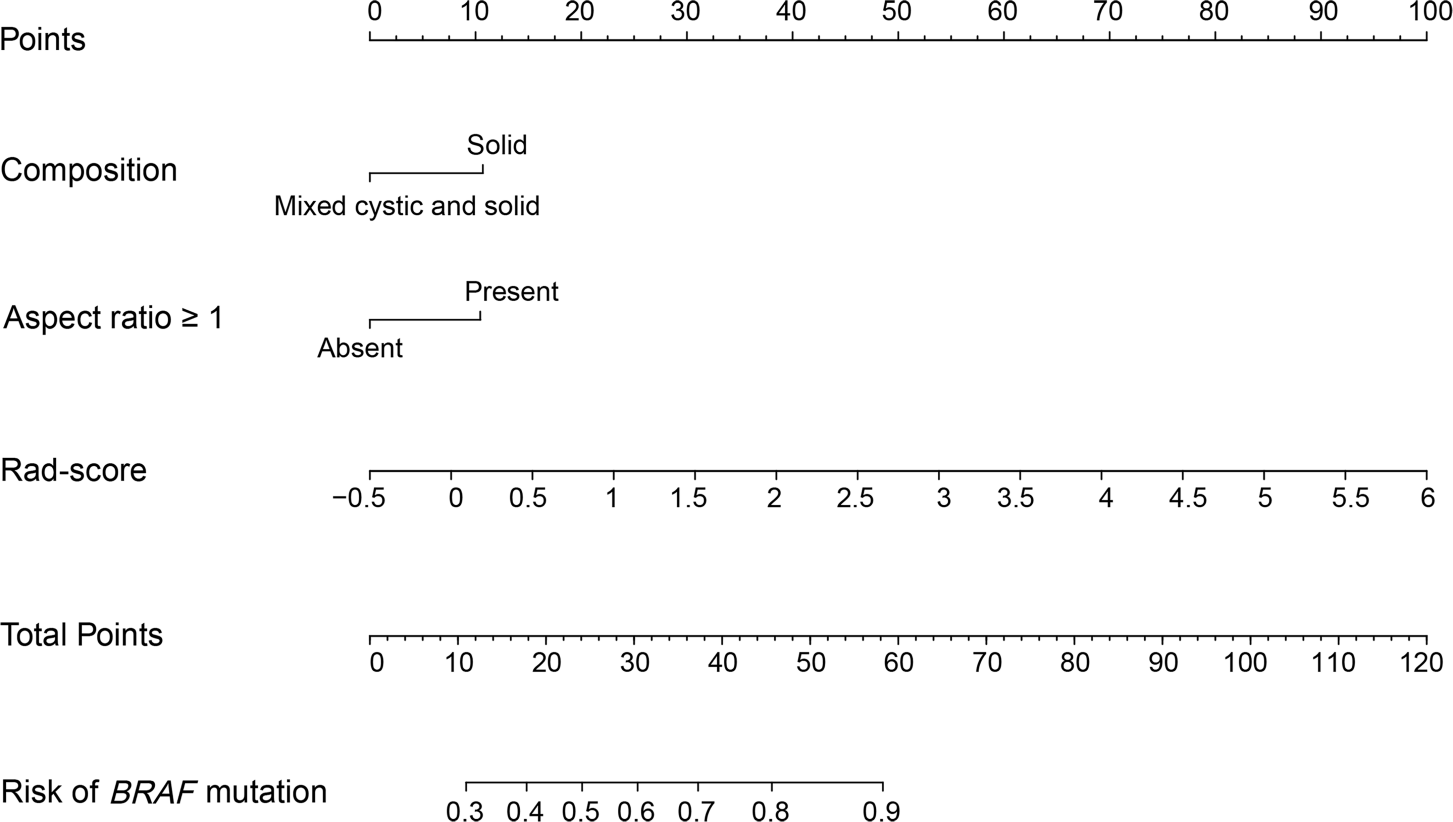
US radiomics nomogram to estimate the risk of *BRAF* mutation in PTMC. US, ultrasound; PTMC, papillary thyroid microcarcinoma; Rad-score, radiomics score.

**Figure 4:**
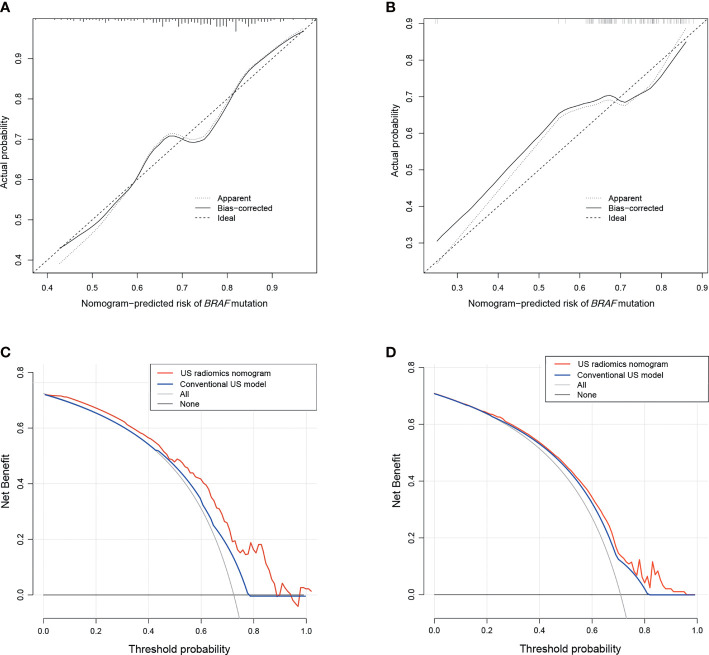
The calibration plots of the US radiomics nomogram for the training **(A)** and the validation cohorts **(B)**. Decision curve analysis of the US radiomics nomogram and conventional US model in the training **(C)** and validation sets **(D)**. US, ultrasound.

The Rad-score, composition, and aspect ratio ≥1 were identified as the three independent predictors of *BRAF* mutation in PTMC patients by a multivariate logistic regression model (**Table 3**). A nomogram for predicting *BRAF* mutation in PTMC was built on the basis of these three key factors (**Figure 3**). The AUCs confirmed a favorable discrimination performance of the nomogram both in the training cohort (AUC = 0.685, 95% CI: 0.610 to 0.760) and the validation cohort (AUC = 0.651, 95% CI: 0.532 to 0.770), which were higher than those of the conventional US model (AUC = 0.592 for the training set, AUC = 0.622 for the validation set). The calibration plots demonstrated optimal consistency between the bootstrap-predicted values and the actual observed values in the training and validation cohorts, indicating the appreciable reliability of the US radiomics nomogram (**Figures 4A, B**). The DCA results showed a wide field of threshold probability, supporting the favorable clinical applicability of the radiomics nomogram in predicting positive *BRAF* mutation for PTMC. Furthermore, the US radiomics nomogram obtained more clinical net benefits than the model based on conventional US features (**Figures 4C, D**).

**Table 3:** Independent predictive factors for *BRAF* mutation of PTMC based on multivariate logistic regression analysis.

	Conventional US model			US radiomics nomogram		
	*β*	Odds ratio (95% CI)	*P*-value	*β*	Odds ratio (95% CI)	*P*-value
Intercept	−0.308			−1.058		
Composition	0.861	2.365 (0.810–6.868)	0.109	0.688	1.990 (0.670–5.859)	0.021
Aspect ratio ≥1	0.705	2.024 (1.079–3.772)	0.027	0.672	1.958 (1.024–3.721)	0.041
Rad-score	NA	NA	NA	0.990	2.691 (1.471–5.313)	0.003

PTMC, papillary thyroid microcarcinoma; CI, confidence interval; Rad-score, radiomics score; NA, not available.

### Predicting *BRAF* mutation based on the Nomo-score

The optimal cutoff value of the Nomo-score was determined to be 1.343. The discrimination and AUC for differentiating the presence of *BRAF* mutation were 0.685 (95% CI: 0.610 to 0.760) in the training cohort and 0.651 (95% CI: 0.532 to 0.770) in the validation cohort, respectively. The performance of the optimal cutoff value of the Nomo-score is summarized in **Table 4**.

**Table 4:** Performance of the prediction Nomo-score for estimating the risk of BRAF mutation.

Value	Training cohort	Validation cohort
Cutoff value	1.343	1.343
Sensitivity	75.6%	55.6%
Specificity	93.8%	94.8%
Positive predictive value	96.9%	86.9%
Negative predictive value	59.4%	75.3%
Diagnostic accuracy	80.6%	78.1%
AUC (95% CI)	0.685 (0.610 to 0.760)	0.651 (0.532 to 0.770)

AUC, area under the receiver operating characteristic curve; CI, confidence interval.

## Discussion

Despite the excellent prognosis for most patients with PTMC, a small percentage of patients potentially experience a more aggressive course of the disease. Some of these patients might choose conservative surveillance rather than surgical treatment because the tumor size is small, and there are no other high-risk clinical manifestations (21). The *BRAF* ^V600E^ mutation has emerged as a promising diagnostic as well as a prognostic indicator of PTC since its initial discovery (6, 22). A close association of *BRAF* ^V600E^ mutation with extracapsular infiltration of PTMC, LN metastasis, and a subsequent higher capsular invasion rate has been reported (23, 24). In that case, preoperative determination of *BRAF* mutation will effectively assist to make more aggressive treatment strategies for high-risk PTMC patients. The cytological examination consisting of FNA and *BRAF* mutation detection was the preferred method to diagnose genotype. Nevertheless, recent guidelines do not recommend FNA and gene detection for patients with a thyroid nodule less than 10 mm (14).

The accurate preoperative identification of *BRAF* mutation could help plan the surgical approach and further improve the outcomes of PTMC patients. Nevertheless, it is difficult to identify *BRAF* mutation using non-invasive methods. Many previous studies have explored the association between US features and *BRAF* mutation status of PTC. Liu et al. found that *BRAF* ^V600E^ mutation was associated with microcalcifications and nodule size after the contrast-enhanced US in PTC (25). Another study showed that a higher ACR TI-RADS score was an independent risk factor for *BRAF* ^V600E^ mutation (26). The centripetal and no significant enhancement were predictive indicators for the presence of *BRAF* mutation (27). Concerning the characteristics of CEUS, one study demonstrated that arrival time and time to peak enhancement in CEUS were notably longer in the *BRAF*-positive group than in the *BRAF*-negative group (15). In summary, the relevant studies based on conventional US features are relatively few and all the conclusions are not consistent. However, due to the insufficiency of related studies and the inconsistent results of previous findings, the relationship between the US characteristics and *BRAF* mutation has not been clear. It is still difficult to predict *BRAF* mutation based on preoperative US features, which may be related to the high dependence on the examiners’ experience.

Radiomics, a recently emerged algorithm to extract quantitative data from image materials, has been utilized to predict the molecular characteristics of various tumors. Yang et al. found that the proposed CT-based radiomics signature was associated with KRAS/NRAS/BRAF mutations in colorectal cancer (16). A subsequent study showed that radiomics texture features could serve as potential biomarkers for determining *BRAF* mutation status and as predictors of 5-year prognostic outcome in patients with advanced-stage colorectal cancer (28). Concerning PTC, in two previous studies, the radiomics method has been proposed based on preoperative US images for evaluating *BRAF* mutation, which demonstrated the limited ability to apply a US radiomics analysis in PTC (29, 30). There are a few points to note about these two studies. Firstly, both of these studies focused on PTC patients and not only on PTMC. Some patients with a large diameter of PTC were originally indicated to undergo surgical resection, whose *BRAF* status did not have much clinical guiding significance. Meanwhile, the conventional US features were not included in these two studies. We believe that the qualitative US features were supposed to be included in the analysis along with quantitative radiomics parameters to improve the diagnostic yield of *BRAF* mutation.

This is the first study to incorporate the radiomics parameters and conventional US features to establish a novel model for predicting *BRAF* mutation in PTMC. The Rad-score, composition, and aspect ratio ≥1 were identified as the three independent predictors of *BRAF* mutation. It was indicated that PTMC patients with a higher Rad-score were more likely to have *BRAF* mutation. In terms of the nodules’ composition, BRAF alteration was more common in solid lesions. This may be due to the fact that, during the FNA procedure, obtaining satisfactory samples to have genetic testing is easier for solid nodules, especially for nodules less than 10 mm. We also found that PTMC with an aspect ratio ≥1 was a predictive sign of *BRAF* mutation. In BRAF-mutated PTC, active extracellular-regulated kinase and active AKT kinase proteins are able to stimulate multiple downstream signaling pathways, which ultimately result in increased proliferation (31). It is speculated that those changes can affect the growth pattern of the tumor, finally leading to the taller-than-wide shape. To provide an easy-to-use tool in clinical practice, we illustrated a US radiomics nomogram based on the three key factors. Our nomogram exhibited good discrimination and calibration in the training and validation sets. The AUC of the nomogram was 0.685 in the training set and achieved higher predictive efficacy than the prediction model involving the conventional US factors alone. DCA demonstrated that the US radiomics nomogram can improve patient *BRAF* prediction preoperatively.

Our study had several limitations. Firstly, this study was a retrospective analysis which might cause a case selection bias. A prospective well-designed study was needed for a more accurate assessment of the relation between the radiomics features and *BRAF* mutations. Secondly, the lack of external validation data was also a limitation of this study. Our results need to be further validated in multicenter investigations to better assess the potential clinical use of the nomogram. Finally, the AUCs of the US radiomics nomogram were relatively low. The reason might be that we only included grayscale US images in the analysis. We will add multimodal US images, including contrast-enhanced and elastography US, to extract more radiomics features in the subsequent study, aiming to improve the predictive ability of the radiomics model.

## Conclusion

Our study indicates that PTMC patients with a higher Rad-score are more likely to present *BRAF* mutations. The nomogram which incorporated US radiomics and conventional US parameters might serve as a potential tool to predict *BRAF* mutation and could assist clinicians to develop individualized strategies for patients with PTMC.

## Data availability statement

The raw data supporting the conclusions of this article will be made available by the authors, without undue reservation.

## Ethics statement

The studies involving human participants were reviewed and approved by Institutional Review Board of China-Japan Friendship Hospital. The patients/participants provided their written informed consent to participate in this study.

## Author contributions

Data curation and writing the original draft: JT. Formal analysis and methodology: SJ. Data curation and validation: JM and XX. Data project administration: HL and LW. Conceptualization, investigation, and funding acquisition: BZ. All authors contributed to the article and approved the submitted version.

## Funding

This study was supported by the China-Japan Friendship Hospital Talent Introduction Project (No. 2019-RC-2) and the National Natural Science Foundation of China (No. 81971627).

## Conflict of interest

The authors declare that the research was conducted in the absence of any commercial or financial relationships that could be construed as a potential conflict of interest.

The reviewer YW declared a shared affiliation, with no collaboration, with one of the authors, SJ, to the handling editor at the time of the review.

## Publisher’s note

All claims expressed in this article are solely those of the authors and do not necessarily represent those of their affiliated organizations, or those of the publisher, the editors and the reviewers. Any product that may be evaluated in this article, or claim that may be made by its manufacturer, is not guaranteed or endorsed by the publisher.

## References

[1] Miranda-Filho A Lortet-Tieulent J Bray F Cao B Franceschi S Vaccarella S. Thyroid cancer incidence trends by histology in 25 countries: a population-based study. Lancet Diabetes Endocrinol (2021) 9(4):225–34. doi: 10.1016/S2213-8587(21)00027-9 33662333

[2] Seib CD Sosa JA. Evolving understanding of the epidemiology of thyroid cancer. Endocrinol Metab Clin North Am (2019) 48(1):23–35. doi: 10.1016/j.ecl.2018.10.002 30717905

[3] Pedrazzini L Baroli A Marzoli L Guglielmi R Papini E. Cancer recurrence in papillary thyroid microcarcinoma: a multivariate analysis on 231 patients with a 12-year follow-up. Minerva Endocrinol (2013) 38(3):269–79. doi: 10.1038/nutd.2013.31 24126547

[4] Noone AM Cronin KA Altekruse SF Howlader N Lewis DR Petkov VI. Cancer incidence and survival trends by subtype using data from the surveillance epidemiology and end results program, 1992-2013. Cancer Epidemiol Biomarkers Prev (2017) 26(4):632–41. doi: 10.1158/1055-9965.EPI-16-0520 PMC5380602 27956436

[5] Zheng X Wei S Han Y Li Y Yu Y Yun X. Papillary microcarcinoma of the thyroid: clinical characteristics and BRAF(V600E) mutational status of 977 cases. Ann Surg Oncol (2013) 20(7):2266–73. doi: 10.1245/s10434-012-2851-z 23370668

[6] Xing M Alzahrani AS Carson KA Shong YK Kim TY Viola D. Association between BRAF V600E mutation and recurrence of papillary thyroid cancer. J Clin Oncol (2015) 33(1):42–50. doi: 10.1200/JCO.2014.56.8253 25332244 PMC4268252

[7] Chen Y Sadow PM Suh H Lee KE Choi JY Suh YJ. BRAF(V600E) is correlated with recurrence of papillary thyroid microcarcinoma: A systematic review, multi-institutional primary data analysis, and meta-analysis. Thyroid (2016) 26(2):248–55. doi: 10.1089/thy.2015.0391 26671072

[8] Walczyk A Kowalska A Kowalik A Sygut J Wypiórkiewicz E Chodurska R. The BRAF(V600E) mutation in papillary thyroid microcarcinoma: does the mutation have an impact on clinical outcome? Clin Endocrinol (Oxf) (2014) 80(6):899–904. doi: 10.1111/cen.12386 24354346

[9] Kim KJ Kim SG Tan J Shen X Viola D Elisei R. BRAF V600E status may facilitate decision-making on active surveillance of low-risk papillary thyroid microcarcinoma. Eur J Cancer (2020) 124:161–9. doi: 10.1016/j.ejca.2019.10.017 31790974

[10] Di Benedetto G Fabozzi A Rinaldi C Rinaldi CR. BRAF test and cytological diagnosis with a single fine needle cytology sample. Acta Cytol (2013) 57(4):337–40. doi: 10.1159/000350618 23860494

[11] Haugen BR Alexander EK Bible KC Doherty GM Mandel SJ Nikiforov YE. 2015 American thyroid association management guidelines for adult patients with thyroid nodules and differentiated thyroid cancer: The American thyroid association guidelines task force on thyroid nodules and differentiated thyroid cancer. Thyroid (2016) 26(1):1–133. doi: 10.1089/thy.2015.0020 26462967 PMC4739132

[12] Shin JH Baek JH Chung J Ha EJ Kim JH Lee YH. Ultrasonography diagnosis and imaging-based management of thyroid nodules: Revised Korean society of thyroid radiology consensus statement and recommendations. Korean J Radiol (2016) 17(3):370–95. doi: 10.3348/kjr.2016.17.3.370 PMC4842857 27134526

[13] Tessler FN Middleton WD Grant EG Hoang JK Berland LL Teefey SA. ACR thyroid imaging, reporting and data system (TI-RADS): White paper of the ACR TI-RADS committee. J Am Coll Radiol (2017) 14(5):587–95. doi: 10.1016/j.jacr.2017.01.046 28372962

[14] Lee JY Baek JH Ha EJ Sung JY Shin JH Kim JH. 2020 imaging guidelines for thyroid nodules and differentiated thyroid cancer: Korean society of thyroid radiology. Korean J Radiol (2021) 22(5):840–60. doi: 10.3348/kjr.2020.0578 PMC8076832 33660459

[15] Chen L Chen L Liu J Nong L Zhang H. The association among quantitative contrast-enhanced ultrasonography features, thyroid imaging reporting and data system and BRAF V600E mutation status in patients with papillary thyroid microcarcinoma. Ultrasound Q (2019) 35(3):228–32. doi: 10.1097/RUQ.0000000000000406 30601445

[16] Yang L Dong D Fang M Zhu Y Zang Y Liu Z. Can CT-based radiomics signature predict KRAS/NRAS/BRAF mutations in colorectal cancer? Eur Radiol (2018) 28(5):2058–67. doi: 10.1007/s00330-017-5146-8 29335867

[17] Gevaert O Echegaray S Khuong A Hoang CD Shrager JB Jensen KC. Predictive radiogenomics modeling of EGFR mutation status in lung cancer. Sci Rep (2017) 7:41674. doi: 10.1038/srep41674 28139704 PMC5282551

[18] Frates MC Benson CB Doubilet PM Cibas ES Marqusee E. Can color Doppler sonography aid in the prediction of malignancy of thyroid nodules? J Ultrasound Med (2003) 22(2):127–31. quiz 132-124. doi: 10.7863/jum.2003.22.2.127 12562117

[19] Szczypiński PM Strzelecki M Materka A Klepaczko A. MaZda–a software package for image texture analysis. Comput Methods Programs BioMed (2009) 94(1):66–76. doi: 10.1016/j.cmpb.2008.08.005 18922598

[20] Sauerbrei W Royston P Binder H. Selection of important variables and determination of functional form for continuous predictors in multivariable model building. Stat Med (2007) 26(30):5512–28. doi: 10.1002/sim.3148 18058845

[21] Oh HS Ha J Kim HI Kim TH Kim WG Lim DJ. Active surveillance of low-risk papillary thyroid microcarcinoma: A multi-center cohort study in Korea. Thyroid (2018) 28(12):1587–94. doi: 10.1089/thy.2018.0263 30226447

[22] Xing M Alzahrani AS Carson KA Viola D Elisei R Bendlova B. Association between BRAF V600E mutation and mortality in patients with papillary thyroid cancer. Jama (2013) 309(14):1493–501. doi: 10.1001/jama.2013.3190 PMC3791140 23571588

[23] Li F Chen G Sheng C Gusdon AM Huang Y Lv Z. BRAFV600E mutation in papillary thyroid microcarcinoma: a meta-analysis. Endocr Relat Cancer (2015) 22(2):159–68. doi: 10.1530/ERC-14-0531 PMC4629836 25593071

[24] Guo L Ma YQ Yao Y Wu M Deng ZH Zhu FW. Role of ultrasonographic features and quantified BRAFV600E mutation in lymph node metastasis in Chinese patients with papillary thyroid carcinoma. Sci Rep (2019) 9(1):75. doi: 10.1038/s41598-018-36171-z 30635590 PMC6329760

[25] Liu Y He L Yin G Cheng L Zeng B Cheng J. Association analysis and the clinical significance of BRAF gene mutations and ultrasound features in papillary thyroid carcinoma. Oncol Lett (2019) 18(3):2995–3002. doi: 10.3892/ol.2019.10641 31452778 PMC6704325

[26] Shangguan R Hu YP Huang J Yang SJ Ye L Lin RX. Association between BRAF(V600E) mutation and the American college of radiology thyroid imaging, reporting and data system in solitary papillary thyroid carcinoma. Acad Radiol (2019) 26(2):154–60. doi: 10.1016/j.acra.2018.05.010 29941398

[27] Lin ZM Yan CX Song Y Hong YR Wen Q Xu YY. The features of contrast enhanced ultrasound and BRAF V600E in papillary thyroid carcinoma. J Thorac Dis (2019) 11(12):5071–8. doi: 10.21037/jtd.2019.11.78 PMC6988045 32030223

[28] Negreros-Osuna AA Parakh A Corcoran RB Pourvaziri A Kambadakone A Ryan DP. Radiomics texture features in advanced colorectal cancer: Correlation with BRAF mutation and 5-year overall survival. Radiol Imaging Cancer (2020) 2(5):e190084. doi: 10.1148/rycan.2020190084 33778733 PMC7983710

[29] Yoon JH Han K Lee E Lee J Kim EK Moon HJ. Radiomics in predicting mutation status for thyroid cancer: A preliminary study using radiomics features for predicting BRAFV600E mutations in papillary thyroid carcinoma. PloS One (2020) 15(2):e0228968. doi: 10.1371/journal.pone.0228968 32053670 PMC7018006

[30] Kwon MR Shin JH Park H Cho H Hahn SY Park KW. Radiomics study of thyroid ultrasound for predicting BRAF mutation in papillary thyroid carcinoma: Preliminary results. AJNR Am J Neuroradiol (2020) 41(4):700–5. doi: 10.3174/ajnr.A6505 PMC7144636 32273326

[31] Kabaker AS Tublin ME Nikiforov YE Armstrong MJ Hodak SP Stang MT. Suspicious ultrasound characteristics predict BRAF V600E-positive papillary thyroid carcinoma. Thyroid (2012) 22(6):585–9. doi: 10.1089/thy.2011.0274 PMC3358112 22524468

