# Supplementary material for: Nomogram based on radiomics analysis of ultrasound images can improve preoperative BRAF mutation diagnosis for papillary thyroid microcarcinoma
Source: Front Endocrinol (Lausanne). 2022 Aug 19;13:915135. doi: 10.3389/fendo.2022.915135 (PMC9437521; doi:10.3389/fendo.2022.915135)
Supplement: Supplementary file 1 [file Table_1.docx]

**Supplementary Table 1.** Texture features from MaZda software.

| **Categories** | **Texture** | **Total number** |
| --- | --- | --- |
| **HISTOGRAM** | Mean (histogram’s mean) Variance (histogram’s variance) Skewness (histogram’s skewness) Kurtosis (histogram’s kurtosis) Perc.01% (1% percentile) Perc.10% (10% percentile) Perc.50% (50% percentile) Perc.90% (90% percentile) Perc.99% (99% percentile) | 9 |
| **GRADIENT** | GrMean (absolute gradient mean) GrVariance (absolute gradient variance) GrSkewness (absolute gradient skewness) GrKurtosis (absolute gradient kurtosis) GrNonZeros (percentage of pixels with nonzero gradient) | 5 |
| **RUN LENGTH MATRIX** | RLNonUni (run length nonuniformity) GLevNonU (grey level nonuniformity) LngREmph (long run emphasis) ShrtREmp (short run emphasis) Fraction (fraction of image in runs) | 20 (2D) or 65 (3D) |
| **COOCURRENCE MATRIX** | AngScMom (angular second moment) Contrast (contrast) Correlat (correlation) SumOfSqs (sum of squares) InvDfMom (inverse difference moment) SumAverg (sum average) SumVarnc (sum ariance) SumEntrp (sum entropy) Entropy (entropy) DifVarnc (difference variance DifEntrp (difference entropy)  Features are computed for 5 between-pixels distances (1, 2, 3, 4, 5) and for 4 (2D images) or 13 (3D images) various directions. | 220 (2D) or 715 (3D) |
| **AUTOREGRESSIVE MODEL** | Teta1 (parametr θ1)  Teta2 (parametr θ2)  Teta3 (parametr θ3)  Teta4 (parametr θ4) Sigma (parametr σ) | 5 |
| **HAAR WAVELET** | WavEn (wavelet energy) Feature is computed at 4 scales within four frequency bands LL, LH, HL and HH. | 16 |
